# Nephroscopic management of partial bladder perforation by an intrauterine device with extravesical main body: a case report and treatment-oriented classification

**DOI:** 10.1186/s12894-026-02256-5

**Published:** 2026-07-13

**Authors:** Haiying Niu, Ruirui Ge, Jiajia Wang, Shijie Yao, Quanxin Qu

**Affiliations:** 1 Department of Gynecology, Tianjin First Central Hospital, 24 Fukang Road, Tianjin, 300192 China; 2 School of Medicine, Nankai University, 94 Weijin Road Nankai, Tianjin, 300071 China; 3 Department of Urology, Tianjin First Central Hospital, 24 Fukang Road, Tianjin, 300192 China

**Keywords:** IUD, Partial perforation, Bladder, Nephroscope

## Abstract

The intrauterine device (IUD) is an important and highly effective means of contraception. Migration of the IUD, post implantation, out of the uterus is an infrequent complication, and an IUD partially perforating the bladder is even more infrequently reported. The authors report a 44-year-old woman who had a copper-M IUD inserted into her uterus 16 years ago. Six months later an unexpected pregnancy occurred and an elective suction abortion was performed without ultrasound check before or after surgery. A copper-T IUD was inserted immediately after completion of the abortion but she was subsequently lost to follow-up. Before detection of the ectopic state of the IUD, she complained of lower quadrant abdominal pain following urination for one month. Physical examination revealed mild tenderness upon deep palpation of the suprapubic area. A series of imaging studies had been performed to confirm the exact position of the ectopic IUD. Transabdominal ultrasound showed a linear hyperechoic foreign body embedded in the right side of the bladder wall, a plain abdominal X-ray showed an M-shaped IUD on the right side of the pelvis, an abdominal CT scan found a portion of one arm penetrated the right anterior bladder wall, and nephroscopy demonstrated a one-centimeter of penetration depth and surrounding calculus formation. The IUD arm was gently pulled into the bladder with forceps and was subsequently removed through the urethra. A Foley catheter was later placed into the bladder for one week to manage the bladder perforation. The patient made an uneventful recovery.

This case demonstrates that partial bladder perforation with the IUD body outside the bladder can be successfully managed via transurethral nephroscopy. A stepwise imaging strategy (ultrasound → KUB → CT) is essential for surgical planning. We propose a treatment-oriented classification and a replicable clinical pathway integrating multidisciplinary collaboration and tailored technology selection.

## Background

Throughout the world, intrauterine contraceptive devices (IUDs) are a popular, reversible, and frequently used contraceptive method without major complications [1]. IUD perforation is a rare complication with an incidence rate of 0.05/1000 -13/1000 [2]. Uterine perforation can be categorized as partial, with part of the device embedded in the uterine wall, or complete, with the device entirely within the abdominal cavity [3]. In contrast, IUD perforation with penetration into the bladder is relatively rare. Due to adhesion formation, calculi development, and other possible complications, the IUD must be completely removed [4]. Therefore, appropriate management of this complication is a clinical priority. Since most IUD migration occurs deep within the body, the symptoms do not help in localization. Therefore, imaging studies guide localization. Clinicians evaluating bladder perforation by an IUD should be prepared for a variety of operative interventions, most involving cystoscopy with or without lithotripsy and less commonly an abdominal approach involving cystotomy and repair.

Herein we report an uncommon complication of an IUD partially perforating the bladder wall with the main part of the IUD outside of the bladder followed by successful removal using a nephroscope.

### Case description

A 44-year-old woman (gravida 3, para 2) with a one-month history of lower quadrant abdominal pain following urination was referred to the urology department. The referral was based on the combination of persistent lower urinary tract symptoms, absence of the IUD strings on pelvic examination, and a history of an undetected IUD during suction abortion 16 years earlier with no documented expulsion. She had no other symptoms such as hematuria or dysuria. The physical examination did not reveal any significant findings, except for mild tenderness upon deep palpation of the suprapubic area. Urine culture was negative.

Sixteen years ago, a copper-M IUD was inserted into the uterus immediately following an uncomplicated vaginal delivery. Six months later an unexpected pregnancy occurred and an elective suction abortion was performed without ultrasound check before or after surgery. The IUD was not identified during surgery and was presumed to have been expelled. A copper-T IUD was inserted immediately after completion of the abortion. The patient was subsequently lost to follow-up.

**Fig. 1:**
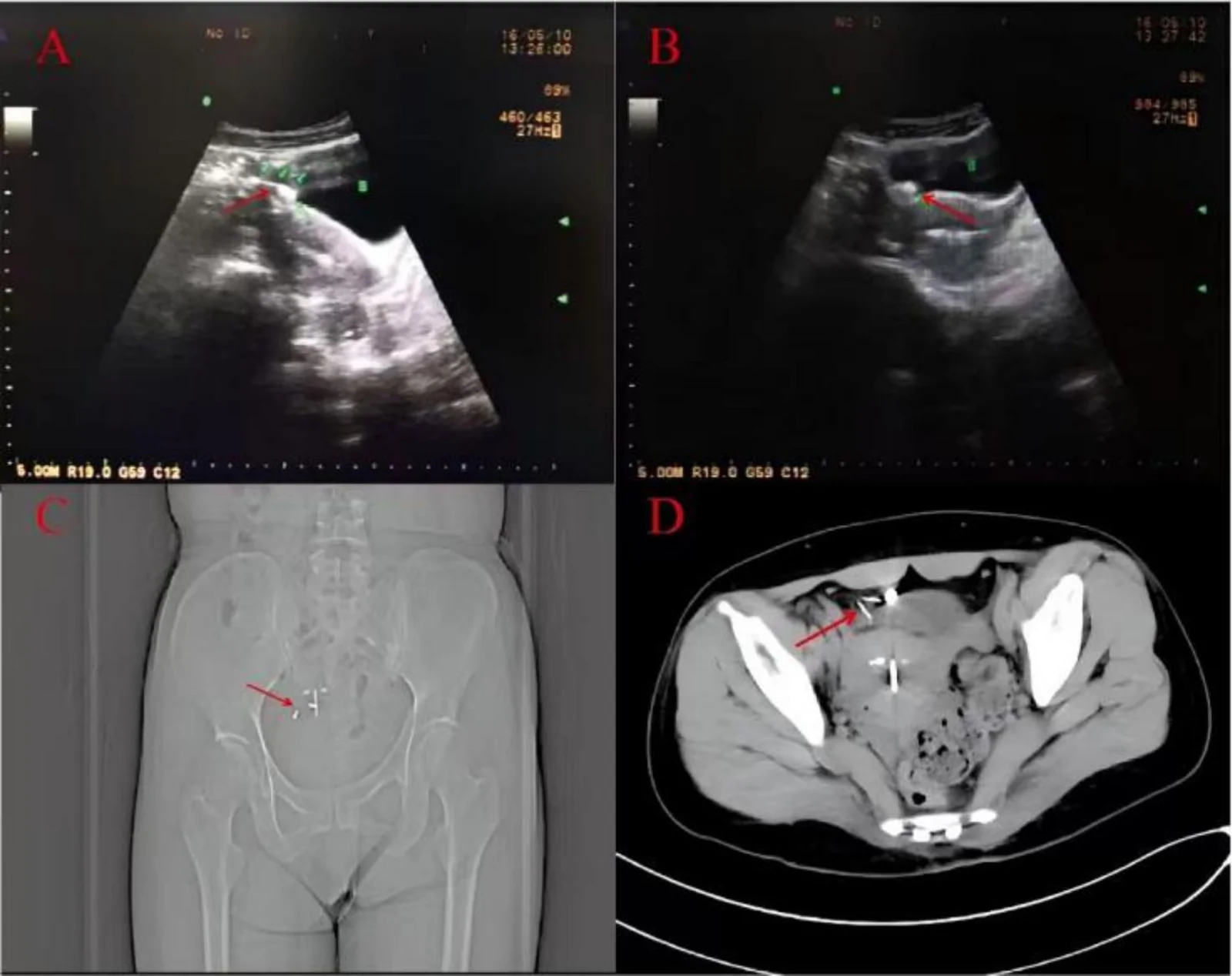
Imaging results. **A** and **B** Ultrasound examination: hard to distinguish whether or not the foreign body was in the bladder cavity (red arrow); **C** Plain abdominal X-ray: a T shaped IUD within the uterine cavity, and an M shaped IUD on the right side of the pelvis (red arrow); **D** Abdominal CT scan: The main part of the M-type IUD was located outside the bladder in the right preperitoneal space, while a portion of one arm penetrated the right anterior bladder wall and extended into the bladder cavity (red arrow)

The urologist ordered a transabdominal ultrasound, which showed a normal sized uterus with a copper T IUD in the normal site and a linear hyperechoic foreign body embedded in the right side of the bladder wall. The ultrasound examination was not helpful in distinguishing whether or not the foreign body was in the bladder cavity because of the superposition of the images (Fig. 1A and B).

Therefore, a plain abdominal X-ray was performed which confirmed the presence of two IUDs in the pelvis. One was a T shaped IUD within the uterine cavity, and the other was an M shaped IUD on the right side of the pelvis (Fig. 1C).

In order to ascertain the relationship of the ectopic IUD with the bladder and its adjacent tissues, an abdominal CT scan was performed. It showed a T-type IUD located in proper position within the uterine cavity and an M-type IUD with a portion of one arm penetrating the right anterior bladder wall and extending into the bladder cavity. The main part of the M-type IUD was located outside the bladder in the right preperitoneal space (Fig. 1D).

**Fig. 2:**
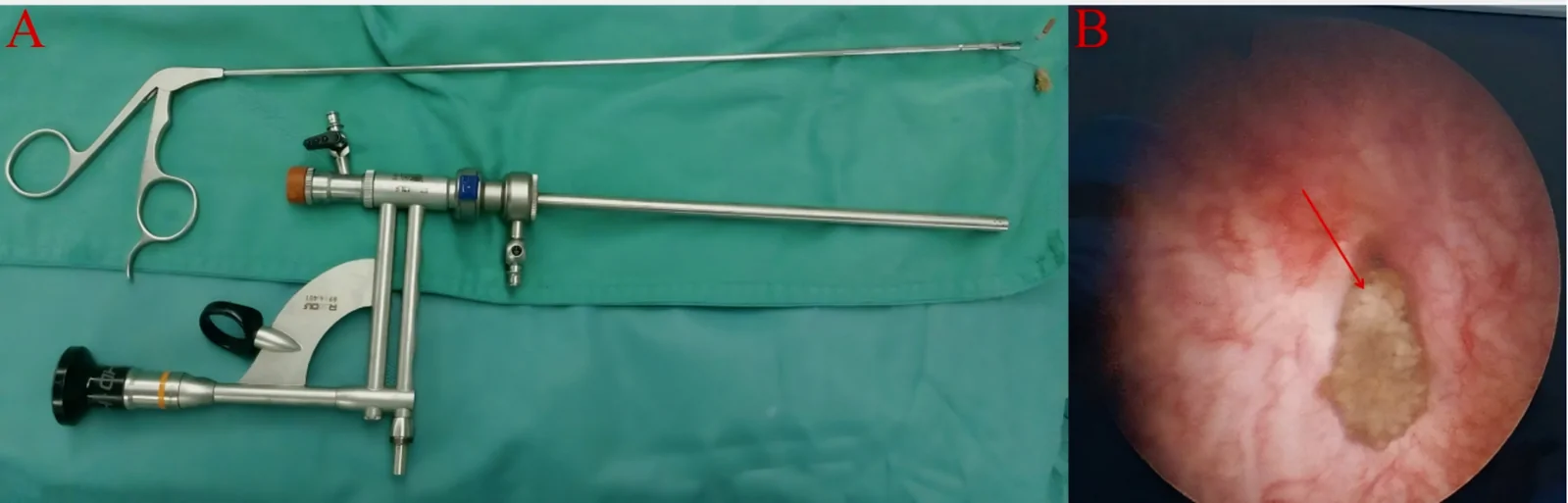
Surgery record. **A** nephroscopy; **B **Nephroscopic view showing the IUD arm penetrating the right anterior bladder wall with surrounding calculus formation (red arrow)

Under general anesthesia, the patient underwent nephroscopy (Fig. 2A), which showed one centimeter of one arm of the M-type IUD had penetrated into the bladder mucosa (Fig. 2B). It was embedded in the right anterior wall of the bladder with surrounding calculus formation .

### Surgical treatment and postoperative follow-ups

**Fig. 3:**
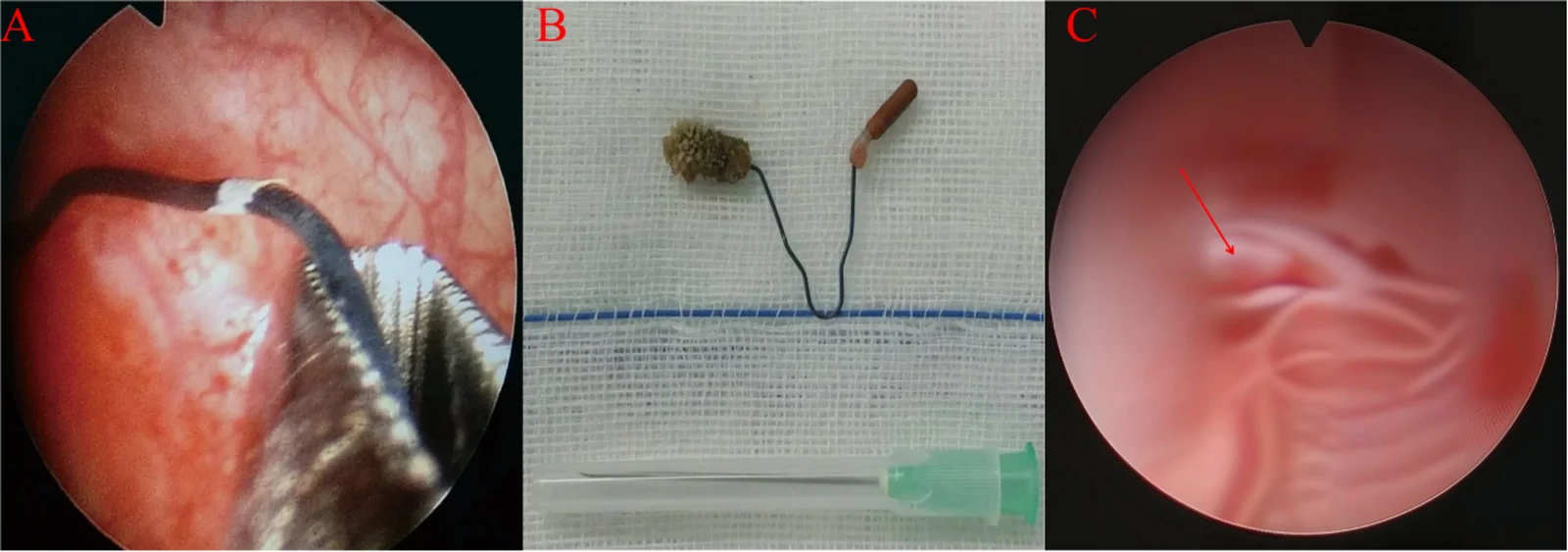
Endoscopic extraction of the IUD and post-removal bladder wall appearance. **A **The IUD arm is grasped with forceps and gently rotated toward the bladder cavity; **B** The intact IUD after complete removal through the urethra; **C** The small shrinkage defect on the inner bladder wall after extraction (red arrow), with no active bleeding or urinary leakage

The intravesical part of the IUD was grasped with forceps. Using gentle traction, the IUD arm was slowly rotated in the direction of the bladder cavity (Fig. 3A) and successfully pulled into the bladder. It was subsequently removed through the urethra intact (Fig. 3B). There was a small mucosal defect on the inner wall of the bladder, but no active bleeding or urinary leakage was noted (Fig. 3C).

To manage the bladder perforation, a Foley catheter was placed into the bladder and removed one week later. The patient had follow-up visits at 4 weeks and 6 months. She had no complaints. Her bimanual exams and pelvic ultrasounds were normal. The treatment procedure was approved by the Ethics Committee and the patient.

## Discussion

The intrauterine device (IUD) is a widely used long-acting reversible contraceptive (LARC) method, known for high efficacy, cost-effectiveness, and user autonomy. Recommended by WHO as a first-line option, it works through endometrial modification, sperm immobilization, and prevention of fertilization with low annual failure rates. Globally, about 13.9% of 1.16 billion reproductive-age women (15–49 years) use IUDs, totaling over 161 million users, and adapt well to diverse healthcare systems. In China, IUD prevalence reaches 40.6% among 269 million women of childbearing age, significantly higher than the global average, due to historical policies, public health availability, cultural acceptance and alignment with women’s need for reliable, low-maintenance contraception [5].

IUD displacement occurs via acute perforation from suboptimal insertion or chronic secondary perforation due to pregnancy, inflammation, or ectopic gestation [6–8]. In our case, conception six months after insertion with the device undetected during curettage suggests pre-existing perforation, followed by gradual migration into the bladder over 16 years. Chronic copper-ion release may have promoted local inflammation and stone formation [9], eventually presenting as post-void abdominal pain.

The symptom of lower abdominal pain after urination served as a critical diagnostic clue, underscoring that in women with a history of IUD placement, lower urinary tract symptoms—including dysuria, frequent urination, gross hematuria, recurrent infections, or new pregnancy after insertion should prompt consideration of IUD migration [10]. What distinguishes this case is the temporal integration of clinical history with presentation. Conception six months after IUD placement, combined with absence of the device during suction curettage, established a clear timeline for perforation. The subsequent 16-year latency before symptom onset illustrates that chronic bladder irritation, rather than acute perforation, may be the predominant manifestation in partial bladder involvement. Three practical implications emerge. First, IUD migration should remain in the differential for lower urinary tract symptoms in women with a history of IUD placement—even without acute pain or hematuria. Second, failure to identify an IUD during pregnancy-related procedures should prompt imaging confirmation, not assumption of spontaneous expulsion [2, 11, 12]. Third, even when a patient reports that the IUD has been removed or assumes it has been expelled, clinicians should maintain a low threshold for radiological confirmation if the device is not visualized during pelvic examination. As Varlas et al. emphasized, the absence of strings does not reliably indicate spontaneous expulsion, and further imaging should be pursued before concluding that the device is no longer in situ [13]. Their review of 115 migrated IUD cases found that many patients were asymptomatic or had vague symptoms, with diagnosis often made incidentally during imaging for unrelated conditions [13]. This underscores that reliance on patient history alone—particularly when the patient is lost to follow-up—carries a substantial risk of missed diagnosis, as clearly illustrated by our case.

Once clinical suspicion is raised, imaging plays a decisive role in confirming the diagnosis and guiding management. A stepwise imaging strategy—ultrasound, followed by plain abdominal radiography (KUB), and then contrast-enhanced computed tomography (CT)—proved to be a replicable and effective diagnostic pathway [14]. In this case, ultrasound revealed a linear hyperechoic structure in the bladder wall but could not distinguish intravesical from extravesical location; KUB confirmed two IUDs; and CT clearly demonstrated partial bladder perforation by the M-shaped IUD arm with the main body in the preperitoneal space. This approach is resource-efficient, progressively narrows the differential, and determines whether endoscopic or abdominal management is feasible. CT is the gold standard for complicated migration, precisely delineating device-organ relationships [14]. Although imaging may occasionally miss migrated IUDs due to calcific artifacts [9, 15, 16], this did not occur in our case.

A recent systematic review of 165 IUD perforation cases reported that bladder displacement accounted for approximately 24% of cases, ranking second after intestinal involvement [17]. Among cases with complete displacement, approximately half were managed by laparoscopy, while other approaches included cystoscopy, laparotomy, and colonoscopy. However, the optimal surgical approach depends on the precise anatomical location of the migrated device. In this case, the stepwise imaging strategy—ultrasound, KUB, and CT—revealed a unique configuration: one arm of the M-shaped IUD partially penetrated the right anterior bladder wall, while the main body lay in the preperitoneal space. This anatomy rendered conventional options suboptimal. Cystoscopy would provide insufficient grasping force for the extravesical component and risks device fragmentation or bladder wall injury. Laparoscopy would require accessing the preperitoneal space with potential bladder injury and greater surgical trauma [18]. Following multidisciplinary discussion, a transurethral nephroscopic approach was selected. The F20 nephroscope offers distinct advantages: a larger working channel, stronger grasping forceps, and controlled rotation under direct visualization [19, 20]. These features enabled secure retrieval of the embedded IUD arm without fragmentation—a critical consideration given the risk of device rupture during extraction [5]. The IUD was successfully removed intact, and the patient recovered uneventfully. This study proposes a classification-based management strategy for partial perforation. Existing classification systems—such as the Zakin classification [21] and the partial-versus-complete dichotomy proposed by Istanbulluoglu et al. [22]—describe anatomical positions but a unified, treatment-oriented grading system tailored specifically for bladder IUD migration remains lacking.

Accordingly, based on the anatomical relationship between migrated IUDs and the bladder wall, combined with surgical difficulty and complication risk stratification, we propose a novel three-tier classification system to standardize clinical management of bladder IUD migration.

Type I (pure intravesical migration) is defined as complete localization of the entire IUD within the bladder lumen. This subtype is optimally managed via minimally invasive cystoscopic removal, which is safe and clinically accessible for uncomplicated cases. However, severe device calcification, extensive encrustation, or dense mucosal adhesion may preclude conventional forceps extraction, requiring adjunctive lithotripsy or alternative surgical strategies [23, 24].

Type II (simple transmural penetration) is characterized by isolated full-thickness penetration of the bladder wall by a portion of the IUD: one segment of the device completely traverses the bladder wall and extends into the bladder lumen, while the major body of the IUD is located either intravesically or extravesically. Notably, the migration is confined solely to the bladder, without adhesion, penetration, or fistula formation involving the uterus, ureters, or intestinal tract. The present case perfectly matches this subtype. Minimally invasive approaches including cystoscopy, nephroscopy, and laparoscopy are the preferred interventions. Endoscopic management avoids laparotomy and minimizes surgical trauma, whereas high-resolution CT is mandatory for preoperative risk screening. Extensive calcification and dense adhesions significantly increase operative difficulty and raise the risks of device fragmentation, incomplete extraction, and iatrogenic bladder injury [19, 25, 26]. Ideal endoscopic candidates are Type II cases with favorable anatomical features, without massive calcification, adjacent visceral involvement, or fistula formation. Nephroscopy exhibits unique superiority in selected cases, with stronger grasping force and a larger working channel than conventional cystoscopy, enabling safer and more efficient device retrieval.

Type III (complex transmural penetration) refers to full-thickness bladder wall perforation accompanied by secondary complicated pathological changes, including vesico-visceral fistulas, multi-organ adhesions, and obscured anatomical planes involving the uterus, ureters, or bowel. Such complex cases generally require laparoscopic or open laparotomy to achieve thorough adhesiolysis, precise tissue repair, and complete device removal. Despite higher invasiveness and prolonged recovery time, transabdominal surgery eliminates the risks of residual device fragments and occult visceral injury associated with pure endoscopic manipulation. The clinical feasibility of each subtype depends on preoperative imaging quality and surgical proficiency [18, 27–29]. Type I and Type II lesions are amenable to minimally invasive endoscopic treatment only when high-risk factors are excluded, while Type III cases routinely require transabdominal intervention. Further large-scale prospective studies are warranted to validate the clinical generalizability of this classification system.

In our case, only minor calculus formation was observed around the penetrated IUD arm, which did not hinder mechanical traction under nephroscopic guidance. However, when extensive calcification or encrustation firmly anchors the device to the bladder wall, simple traction may become difficult or carry a higher risk of mucosal injury or device fragmentation. In such scenarios, holmium: YAG laser lithotripsy offers a valuable adjunctive or alternative approach. As demonstrated by Torres Zapata et al., this technique allows for safe fragmentation of adherent calcifications under direct vision, facilitating IUD release while minimizing thermal injury to the surrounding bladder mucosa [30]. Although not required in our patient due to the limited extent of calcification, this endoscopic modality should be considered in cases where calcification precludes straightforward removal, thereby further expanding the minimally invasive armamentarium for managing migrated IUDs with bladder involvement.

Beyond calcification, another critical consideration when planning a purely endoscopic extraction is the potential for unrecognized adhesions or fistulas between the IUD and adjacent bowel. Gong and Shen reported a case where a vesicoenteric fistula only became clinically apparent two months after cystoscopic IUD removal, ultimately requiring intestinal resection [31]. Campobasso et al. similarly described extensive adhesions between a partially intravesical IUD and a small bowel loop discovered during laparoscopic exploration, which would have been missed by a purely endoscopic approach [32]. These observations indicate that the absence of bowel symptoms or overt fistula on preoperative imaging does not reliably exclude intestinal involvement. Liu and Dong further noted that when an IUD is partially located outside the bladder with surrounding adhesions, laparoscopy enables direct visualization of the IUD-organ relationship and reduces the risk of occult injury [33]. Heaney et al. demonstrated that robot-assisted laparoscopy offers enhanced precision for cystotomy and repair in such complex scenarios [34]. Therefore, while a transurethral nephroscopic approach was appropriate for our patient based on favorable anatomy, the decision for a purely endoscopic route must be individualized. When imaging or intraoperative findings suggest bowel adherence or deep transmural involvement, a laparoscopic or robot-assisted approach—alone or combined with endoscopy—should be strongly considered to permit complete assessment and safe extraction.

Additionally, intraoperative fluoroscopy was not utilized in this case, as the IUD arm was directly visible via nephroscopy and its spatial relationship to the bladder wall had been precisely defined by preoperative CT. However, in cases where the device is not endoscopically visible or has fragmented during retrieval, real-time X-ray guidance may serve as a useful adjunct for localization and complete extraction.

In terms of postoperative management, postoperative cystography was not performed before catheter removal because the bladder defect was small, no intraoperative leakage was observed, and the patient remained asymptomatic during catheterization. This approach is consistent with the principle that routine cystography can be safely omitted in selected patients with small, watertight bladder repairs, reserving it for cases with large defects, intraoperative leakage, or postoperative symptoms suggestive of extravasation.

The successful management of this case benefited from multidisciplinary collaboration between the Departments of Urology and Gynecology. This integrated approach aligns with recent literature emphasizing the importance of multidisciplinary coordination in complex IUD migration cases [9]. Liu et al. similarly reported that successful management of partial bladder perforation required coordinated preoperative planning and combined endoscopic execution by gynecologic and urologic teams. This collaborative framework was structured around three phases that align with the stepwise strategy outlined above: preoperative imaging to define anatomy, intraoperative synergy combining urological endoscopic expertise with gynecological knowledge of pelvic anatomy, and postoperative follow-up to monitor for complications from both specialties. This integrated approach not only enables the safe and complete retrieval of the migrated IUD while minimizing surgical trauma, but also offers a reproducible clinical pathway for similar cases of complex IUD migration, particularly those involving partial bladder perforation with the main body outside the bladder .

## Conclusion

This case demonstrates that partial bladder perforation by an IUD with the main body located outside the bladder—a rare and diagnostically challenging condition—can be successfully managed via a transurethral nephroscopic approach. The stepwise imaging strategy (ultrasound → KUB → CT) proved essential for accurate preoperative assessment and surgical planning. Based on this experience, we propose a treatment-oriented classification for partial bladder perforation and a replicable clinical pathway integrating multidisciplinary collaboration and technology selection tailored to device configuration. For carefully selected cases, nephroscopy offers a minimally invasive alternative to conventional laparoscopy or laparotomy, expanding the therapeutic options for this uncommon complication.

## Data Availability

No datasets were generated or analysed during the current study.
